# Nitrous Oxide-Induced Neuropathy among Recreational Users in Vietnam

**DOI:** 10.3390/ijerph18126230

**Published:** 2021-06-09

**Authors:** Xuan Thi Dang, Thanh Xuan Nguyen, Thu Thi Hoai Nguyen, Hung Tran Ha

**Affiliations:** 1 Vietnam Poison Control Center, Bach Mai Hospital, Hanoi 100000, Vietnam; hatranhung@hmu.edu.vn; 2 Department of Emergency and Critical Care Medicine, Hanoi Medical University, Hanoi 100000, Vietnam; 3 Geriatrics Department, Hanoi Medical University, Hanoi 100000, Vietnam; xuanthanh1901vlk@gmail.com (T.X.N.); nththu.bvlk@gmail.com (T.T.H.N.); 4 Scientific Research Department, National Geriatric Hospital, Hanoi 100000, Vietnam; 5 Dinh Tien Hoang Institute of Medicine, Hanoi 100000, Vietnam

**Keywords:** N_2_O, nitrous oxide, nitrous oxide toxicity, Vietnam, neurology

## Abstract

Nitrous oxide (N_2_O) commonly referred to as laughing gas, has significant medical uses. This study aims to describe the neurological disorders associated with N_2_O. We conducted across-sectional study that enrolled patients with nitrous oxide toxicity admitted to Vietnam Poison Control Center, Bach Mai Hospital, Hanoi, Vietnam from June 2018 to July 2019. The questionnaire included demographic characteristics, characteristics of using N_2_O, signs and clinical symptoms, neuroimaging findings, injury on electromyography (EMG) and the Total Neuropathy Score clinical version (TNSc) criteria. A total of 47 participants were included with mean age: 24.38 ± 6.20 years. The number of balloons used per week was 130.59 ± 117.43. The mean duration of N_2_O exposure was 8.79 ± 7.1 months. Multivariate linear logistic regression revealed that the number of N_2_O balloons used per week was significantly associated with TNSc point (Beta: 0.315; 95% CI: 0.001–0.022). We found that myeloneuropathy and peripheral neuropathy were the main neurological disorders related to N_2_O abuse, which should improve the awareness of the appearance of neurological disorders associated with N_2_O abuse.

## 1. Introduction

Nitrous oxide (N_2_O), a colourless non-flammable gas has significant medical uses, especially in surgery and dentistry, because of its anaesthetic and pain reducing effects [1,2]. It is commonly referred to as laughing gas because it producesa feeling of intoxication when inhaled. N_2_O was consumed via gas-filled balloons, at clubs and festivals where the use of recreational drugs is popular.

In recent years the recreational use of inhaled nitrous oxide gas has becomeincreasingly popular, with the United Kingdom logging the highest prevalence of use, followed by the United States (38.6% and 29.4% lifetime prevalence, respectively) [3]. No comprehensive data exist regarding the prevalence and demographics of nitrous oxide misuse in Vietnam. However, the substance has been involved in numerous drug incidents in Vietnam, some deadly. In 2018, seven people died of overdoses at a Hanoi electronic music festival, prompting authorities to suspend all music festivals in the capital until further notice. The police confirmed that the attendees who died had used balloons containing N_2_O. N_2_O is currently being abused across multiple populations and represents a significant public health problem.

Side effects of long-term nitrous oxide use can vary [4]. The most common side effects are neurologic disorders, including myeloneuropathy, subacute combined degeneration, peripheral neuropathy or polyneuropathy, and myelopathy [5,6]. Other less common manifestations of toxicity include frostbite of the mouth, urinary retention, painful erection, decreased libido, delusions, paranoia, anxiety, and even death [4,7].

Although supplying nitrous oxide for recreational purposes is illegal in some countries, its widespread use in the food industry makes its control almost impossible [7]. Currently, Vietnam does not regulate nitrous oxide usage, only its sale and production. Policy makers in Vietnam are looking to categorize laughing gas as an illegal narcotic and to introduce a ban on the substance. The literature shows that neurological disorders are associated with N_2_O abuse, but there are few studies relating to neurological disorders being caused by the recreational use of N_2_O in Vietnam. Therefore, we have undertaken this study to describe the neurological disorders associated with N_2_O abuse among recreational users in Vietnam.

## 2. Materials and Methods

### 2.1. Study Setting and Participants

We conducted a cross-sectional study on inpatients with N_2_O toxicity who were admitted to the Vietnam Poison Control Center, Bach Mai Hospital, Hanoi, Vietnam from June 2018 to July 2019. This center is a referral government hospital and is the only poison control center (PCC) in the north of Vietnam.

The patients received a clinical evaluation that included a medical history evaluation, a physical examination, and a neurologic examination to test for levels of vitamin B12 and homocysteine. All patients received electromyography (EMG) and magnetic resonance imaging (MRI).

Patients were included if they met criteria 1 and one of criteria 2, 3, and 4: (1) using N_2_O; (2) the presence of mental and neurological disorders; (3) a motor function disorder; and (4) subclinical characteristics, including increased homocysteine levels or abnormal results on EMG and MRI.

Patients were excluded from the study if they had at least one of the following criteria: (1) a lack of signs or symptoms of N_2_O toxicity after an observation period; (2) a history of neurological diseases before using N_2_O; (3) a refusal to participate in the study.

A total of 47 patients were enrolled in the study.

### 2.2. Measures and Instruments

After obtaining consent, data were collected using a pre-designed and structured questionnaire by interviewing patients and their families, and by conducting a clinical evaluation at the time of admission. The questionnaire included the following:

### 2.3. Demographic Characteristics: Age and Gender

Characteristics of using N_2_O: history of inhaled N_2_O (balloons), N_2_O exposure time (months), number of balloons used per week (balloons), symptom onset (months) and place of using N_2_O.

Signs and clinical symptoms: neurologic symptoms (sensory deficit or reduced pin sensation, whereby a safety pin was used to lightly prick the face, torso, and 4 limbs; the patient was asked whether the pinprick felt the same on both sides and whether the sensation was dull or sharp), vibration sensory impairment, weakness, dizziness/headache, decreased/loss of a deep tendon reflex (the total number who had any reduction/loss of either upper or lower deep tendon reflexes), decreased/loss of upper limb’s deep tendon reflex, decreased/loss of lower limb’s deep tendon reflex, decreased/loss of upper muscle power, decreased/loss of lower muscle power, Lhermitte’s sign, Romberg’s sign, memory impairment (patient self-reported and interviewed their families), autonomic dysfunction (autonomic dysfunction was determined if subjects had at least 1 symptom including syncope, diarrhea, constipation, bowel or bladder incontinence, or sexual failure), Babinski’s sign, psychiatric symptoms (depression, insomnia, hallucination), and other symptoms (constipation, urinary incontinence, or sexual dysfunction).

Neuroimaging results: MRI injury of the spinal cord (cervical spinal cord and thoracic spinal cord), number of injury spinal segments on MRI (≤7 and >7), and spinal MRI changes (T1W and T2W).

Injury on EMG of the upper limbs (myelin, axon and mix) and injury on EMG lower limbs (myelin, axon and mix), based on electromyography and nerve conduction studies, was performed by neurologists.

Blood tests: hemoglobin (normal: 110–150 g/dL and anemia <110 g/dL), vitamin B12 (normal: 180–910 pg/mL and decreased <180 pg/mL) and homocysteine (normal: 0–20 μmol/L and increased >20 μmol/L).

Urine drug screening was used to evaluate drug abuse in patients.

Chemotherapy-induced peripheral neurotoxicity condition was diagnosed using the total neuropathy score clinical version (TNSc) criteria. The criteria included 7 domains (motor, pin-rick, sensory, vibration, deep tendon reflex, autonomic, and strength) with 0–4 scored for each. Total scores ranged from 0 to 28 points. Total scores from 1–7 points were mild level, 8–14 points were moderate, 15–21 points were severe, and over 21 points were very severe [8].

### 2.4. Data Analysis

All of the data were recorded and analyzed using the Statistical Package for the Social Sciences (SPSS, IBM Corporation, Armonk, NY, USA), version 22.0. Descriptive statistics for the participant characteristics were presented as mean +/− SD for the continuous variables and frequency/percentages for the categorical variables.

Multivariate linear logistic regression was used to identify associated factors with TNSc score. Standardized beta coefficients were presented along with the *p*-value and 95% confident intervals. A *p*-value of less than 0.05 was recognized as statistically significant.

### 2.5. Ethics Approval and Informed Consent

The study was approved by the Ethics Committee of Bach Mai Hospital. Written in-formed consent was obtained from all participants. For patients who were not of legal age to sign on their own behalf, written informed consent was obtained from their guardians. The questionnaire did not record private information, nor was any content that would be harmful to the participants. The results of the research were used only for study purposes. We can confirm that our study also complied with the Declaration of Helsinki.

## 3. Results

A total of 47 participants were included in the study. At the time of admission, all patients had a negative drug abuse test. N_2_O abuse occurred most frequently among adults (mean age of 24.38 ± 6.20 years). The number of balloons used per week were 130.59 ± 117.43. The mean duration of N_2_O exposure was 8.79 ± 7.1 months and the mean time from the last N_2_O exposure to the onset of symptoms was 8.46 ± 6.96 days (Table 1).

The most common neurological symptoms and signs were abnormal sensory symptoms (100%) with reduced pin sensation (100%) and weakness (lower limb superiority). Moreover, vibration sense impairment was also found among a considerable number of patients (74.47%). Tendon reflex abnormalities were also found; 85.1% showed a decreased/loss of lower deep tendon reflex, and 78.7% showed a positive Romberg’s sign. (Table 2).

Psychiatric symptoms were insomnia (4 cases, 8.5%) and depression (1 cases, 2.1%), while no patient presented with the symptom of hallucinations. Memory impairment, urinary incontinence, digestive disorder, and sexual dysfunction were found in 31.9%, 6.4%, 4.3%, and 2.1 % of the participants, respectively. The mean total neuropathy score was 12.61 ± 4.17, which indicated a moderate level of severity (Table 2).

The main abnormal findings in the blood tests were vitamin B12 deficiency in 40.4% of the patients, hyper-homocysteine in 85.1% of the patients, and anemia in 12.8% (Table 2).

The cervical spinal cord was more frequently impaired than other areas of the spinal cord; 27 patients of the 47 (57.4%) showed lesions in the cervical spinal cord, 4 patients (8.5%) showed lesions in the thoracic spinal cord, and 1 patient showed lesions in both the cervical and thoracic spinal cord (Table 3).

A total of 31 patients had MRI changes in the spine; 22 (71%) had T2 hyperintensity of posterior column, while 100% of the damage on T1W was linked to hypointensity or isointensity. The majority of patients with abnormal MRI findings showed lesions with less than seven spinal segments (mean spinal segments injure: 5.24 ± 2.10) (Table 3).

Electromyography showed both myelin and axon impairment in all of the patients. Myelin impairment was the typical electromyography finding (93.6%) and was more prominent in the lower limbs (Table 3).

Multivariate linear logistic regression revealed that the number of N_2_O balloons used per week was significantly associated with the TNSc score (Beta: 0.315; 95% CI: 0.001–0.022) (Table 4).

## 4. Discussion

The study showed that sensory deficits, reduced pin sensation, vibration sense impairment and distal limb weakness, Romberg’s sign, and decreased deep tendon reflex were the most common clinical symptoms of neurological disorders related to N_2_O abuse. Less common neurological signs and symptoms at the time of presentation include memory impairment, bowel and bladder dysfunction, sexual dysfunction, dizzy/headache, Lhermitte’s sign, and psychiatric symptoms. All lesions found were hyperintense on T2W and located in the posterior column. Myelin impairment was the typical electromyography finding. Our findings are similar to those reported in other studies [4,6,9,10,11,12].

A variety of mechanisms are responsible for N_2_O’s neurotoxic effects [13]. The main mechanism of neurological disorders caused by N_2_O abuse is its interference with vitamin B12 metabolism [14]. Vitamin B12 is an essential cofactor for methionine synthase and participates in the synthesis of nucleic acids, carbohydrates, and lipids, which are crucial to the production and maintenance of myelin. N_2_O irreversibly inactivates vitamin B12 by oxidizing its cobalt moiety from the Co^1+^ to the Co^3+^ valence state, leading to demyelination in the central and peripheral nervous system [15]. Vitamin B12 deficiencies can have a variety of causes but are mainly due to limited dietary intake or malabsorption of the vitamin [16]. The prevalence of vitamin B12 deficiency among N_2_O abuse in our study was higher (40%) than that of subjects of the same age in other studies [17]. Scalabrino et al. proposed some key aspects of the pathogenesis of cobalamin deficient neuropathy induced in the central nervous system, particularly in the spinal cord [18]. They demonstrated that the myelin lesions of the cobalamin-deficient neuropathy, whose hallmark is intramyelinic edema in the central nervous system white matter, are caused by locally increased production of a neurotoxic agent, tumor necrosis factor, together with the locally decreased production of a neurotrophic agent, epidermal growth factor. The posterior column of the spinal cord, which plays a crucial role in deep sensory conduction, including the sensory perception of position and vibration [19], is the most common injury location associated with N_2_O intoxication.

In addition, N_2_O itself can produce neurotoxicity by inhibiting the synthesis and release of xanthine and monoamine (dopamine, norepinephrine, and serotonin), thus affecting the cytokine balance and inducing cerebral hypoxia and acidosis [10]. This can be one of the causes of neurologic changes from N_2_O inhalation, resulting in psychosis.

The literature suggests that N_2_O-related neurological disorders are generally associated with intensive and long-term use [20]. Our study found that the number of N_2_O balloons used per week was a significant factor associated with the increase in the TNSc point. This is consistent with the results of a study of 16,239 individuals who had used nitrous oxide in the last 12 months: the greater the dose per session, the greater the predicted probability of reporting paraesthesia [5].

There are some limitations to this study. First, it is difficult to truly and accurately quantify the degree of N_2_O abuse. Participants may be less forthcoming about their amount and duration of use. We also did not determine the exact amount of N_2_O in each balloon. Second, this study used a small sample. We recommend that further studies should be conducted with a larger sample size and in multiple hospitals.

This study has several implications. While the majority of research in the world is retrospective, our research design has overcome the shortcomings with a predetermined protocol. Nitrous oxide-induced neuropathy in the study was comprehensively evaluated through clinical examination and blood tests, as well as functional exploration.

## 5. Conclusions

In conclusion, we found that myeloneuropathy and peripheral neuropathy were the main types of neurological disorders related to N_2_O abuse, which should improve the awareness of the appearance of neurological disorders associated with N_2_O abuse.

## Figures and Tables

**Table 1:** Demographic of the patients, nitrous oxide abuse and amounts used (*n*= 47).

Characteristics	Mean (SD)/*n* (%)	Range
Age	24.38 (6.20)	15–50
Gender		
Male	22 (46.8)	
Female	25 (53.2)	
History of inhaled N_2_O (balloons)		
N_2_O exposure time (months)	8.79 (7.1)	2–36
Number of balloons used per week (balloons)	130.59 (117.43)	12–480
Symptom onset (months)	8.46 (6.96)	1.5–32
Place of using N_2_O		
Home	11 (23.4)	
Bar	29 (61.7)	
Public place	7 (14.9)	

**Table 2:** Clinical characteristics and laboratory findings (*n* = 47).

Presenting Symptoms	*n*	%
Neurologic symptoms and signs		
Sensory deficit	47	100
Reduced pin sensation	47	100
Weakness	41	87.2
Vibration sense impairment	35	74.47
Decreased/Loss muscle power lower	37	78.7
Decreased/Loss muscle power upper	11	55.3
Decreased/Loss deep tendon reflex	40	85.1
Decreased/Loss lower limbs’ deep tendon reflex	38	80.8
Decreased/Loss upper limbs’ deep tendon reflex	26	55.3
Romberg sign (+)	37	78.7
Memory impairment	15	31.9
Dizzy/headache	2	4.3
Ihermitt sign (+)	12	25.5
Autonomic dysfunction	7	14.9
Babinski sign (+)	5	10.6
Psychiatric symptoms		
Depression	1	2.1
Insomnia	4	8.5
Hallucination	0	0.0
Other symptoms		
Constipation	2	4.3
Urinary incontinence	3	6.4
Sexual dysfunction	1	2.1
Laboratory findings		
Hemoglobin(normal 110–150 g/dL)		
Normal	41	87.2
Anemia	6	12.8
Vitamin B12 (normal 180–910 pg/mL)		
Normal	28	59.6
Decreased	19	40.4
Homocysteine (normal 0–20 μmol/L)		
Normal	7	14.9
Increased	40	85.1

**Table 3:** MRI and electromyography data of N_2_O abuse patients included in this study (*n* = 47).

MRI Injury of the Spinal Cord (*n* = 31)		
Cervical spinal cord (Yes)	27	57.4
Thoracic spinal cord (Yes)	4	8.5
Spine MRI changes (*n* = 31)		
T1W		
Hypointensity	8	25.8
Isointensity	23	74.2
Hyperintensity	0	0
T2W		
Hyperintensity	31	100
Posterior column injury	22	71
Number of injury spinal segments on MRI (*n* = 31)		
Mean ± SD	5.24 ± 2.10	
≤7	30	96.8
>7	1	3.2
Injury on electromyography in upper limbs (*n* = 47)		
Myelin	33	70.2
Axon	7	14.9
Mix	7	14.9
Injury on electromyography in lower limbs (*n* = 47)		
Myelin	44	93.6
Axon	30	63.8
Mix	30	63.8

**Table 4:** Multiple Linear logistic between TNSc point with related factors.

Characteristics	Beta	95% CI	*p*-Value
Gender	0.211	−0.662–4.134	0.152
Time of using N_2_O (month)	0.175	−0.083–0.296	0.264
Number of balloons per week	0.315	0.001–0.022	0.047

## Data Availability

The data presented in this study are available on request from the corresponding author.
